# Indoor and outdoor NO_2_ and SO_2_ levels in 13 randomly selected preschools from 7 districts in Mpumalanga Province, South Africa

**DOI:** 10.1002/puh2.175

**Published:** 2024-05-08

**Authors:** Mudau Rodney, Voyi Kuku, Shirinde Joyce

**Affiliations:** ^1^ Department of Human Nutrition, Faculty of Health Sciences University of Pretoria Pretoria South Africa; ^2^ School of Health Systems and Public Health, Faculty of Health Sciences University of Pretoria Pretoria South Africa

**Keywords:** indoor environment, NO_2_, outdoor environment, preschools, SO_2_

## Abstract

**Background:**

Air pollution is a serious worldwide issue, where both outdoor and indoor air quality have a substantial impact on the health of children. Children are more vulnerable to the effects of air pollution due to their developing respiratory systems and higher respiration rates than adults. These children dedicate a substantial amount of time to the preschool setting. The study sought to evaluate the concentration of NO_2_ and SO_2_ in both outdoor and indoor environments of preschool facilities.

**Methods:**

Concurrently, the levels of NO_2_ and SO_2_ were measured indoors and outdoors at 13 randomly selected preschools in 7 districts in Mpumalanga Province, South Africa. Radiello passive air samplers were employed to collect gaseous samples.

**Results:**

The mean levels of NO_2_ indoors and outdoors were within the recommended criteria outlined by the World Health Organization (WHO), specifically target levels 1–3. Additionally, these values were also below the air quality guides outlined by South Africa, both for yearly and 1‐h mean measurements. Nevertheless, both levels remained above the suitable threshold of 10 µg m^−3^ outlined by the WHO air quality guidelines.

**Conclusions:**

It is important to implement proactive measures such as enhancing airflow systems, employing air filters and reducing potential sources of air pollution in preschools, to aid in reducing exposure to indoor and outdoor NO_2_ and SO_2_.

## Graphical abstract

Indoor and outdoor NO_2_ mean levels in South African preschools were within WHO's target levels 1–3, below South Africa's air quality guides but both levels were above WHO's 10 µg m^−3^ threshold. Proactive measures needed: enhance airflow systems, use air filters and reduce pollution sources in preschools.

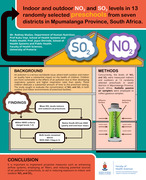

## BACKGROUND

Air pollution is a major global concern, with outdoor and indoor air quality playing a significant role in children's health. The World Health Organization (WHO) reports that over 7 million individuals worldwide die annually due to the detrimental effects of substandard air quality [1]. Children are especially susceptible to the impacts of air pollution because of their growing respiratory systems and higher rates of respiration compared to adults [2].

In Africa, air pollution is a growing problem, with a significant impact on public health. A survey indicated that in 2019, air pollution was responsible for around 1.1 million fatalities in Africa, with 63% of these deaths attributed to exposure to residential air pollution [3]. The rapid urbanisation in African cities is drastically increasing air pollution and greenhouse gas emissions, making it a significant concern for the continent [4].

Air pollution is a significant concern in South Africa, where nearly the entire population is exposed to air that fails to satisfy the standards set forth by the WHO [5]. The country's longstanding dependence on coal for electricity generation has contributed to high levels of air pollution, particularly in industrialized areas such as the Highveld [6]. The main sources of air pollution in South Africa include industry, thermal power stations, smelters and transportation emissions [7].

Exposure to air pollution can have serious health effects on children, including respiratory and cardiovascular illnesses, and impaired lung function [1]. Moreover, poor air quality can also impact cognitive ability in young children [2]. In a study conducted in Sweden, it was found that nasal patency was decreased in schools with lower air exchange rates, and there were more nasal symptoms in schools with more settled dust [8]. Exposure to high levels of both NO_2_ and SO_2_ concurrently increased the risk of developing asthma during the initial year of life [9]. The time children spend in the school environment varies, but they are often at greater risk of exposure to air pollution due to their unique activity patterns and behaviour, as well as the time they spend indoors and outdoors [10]. This exposure can have significant effects on their health, making it important to address air quality concerns in and around school environments [10].

Few studies have measured indoor and outdoor air quality when assessing the relationship between classroom environment (more especially SO_2_ and NO_2_) and the risk of developing asthma or its symptoms in preschool children. Therefore, the paucity of studies in this particular field necessitates the need for the assessment of Indoor and outdoor SO_2_ and NO_2_ levels in 13 randomly selected preschools from 7 districts in Mpumalanga, Province, South Africa. The correlation between the two pollutants was also compared.

## METHODS

### Study design and study setting

**FIGURE 1:**
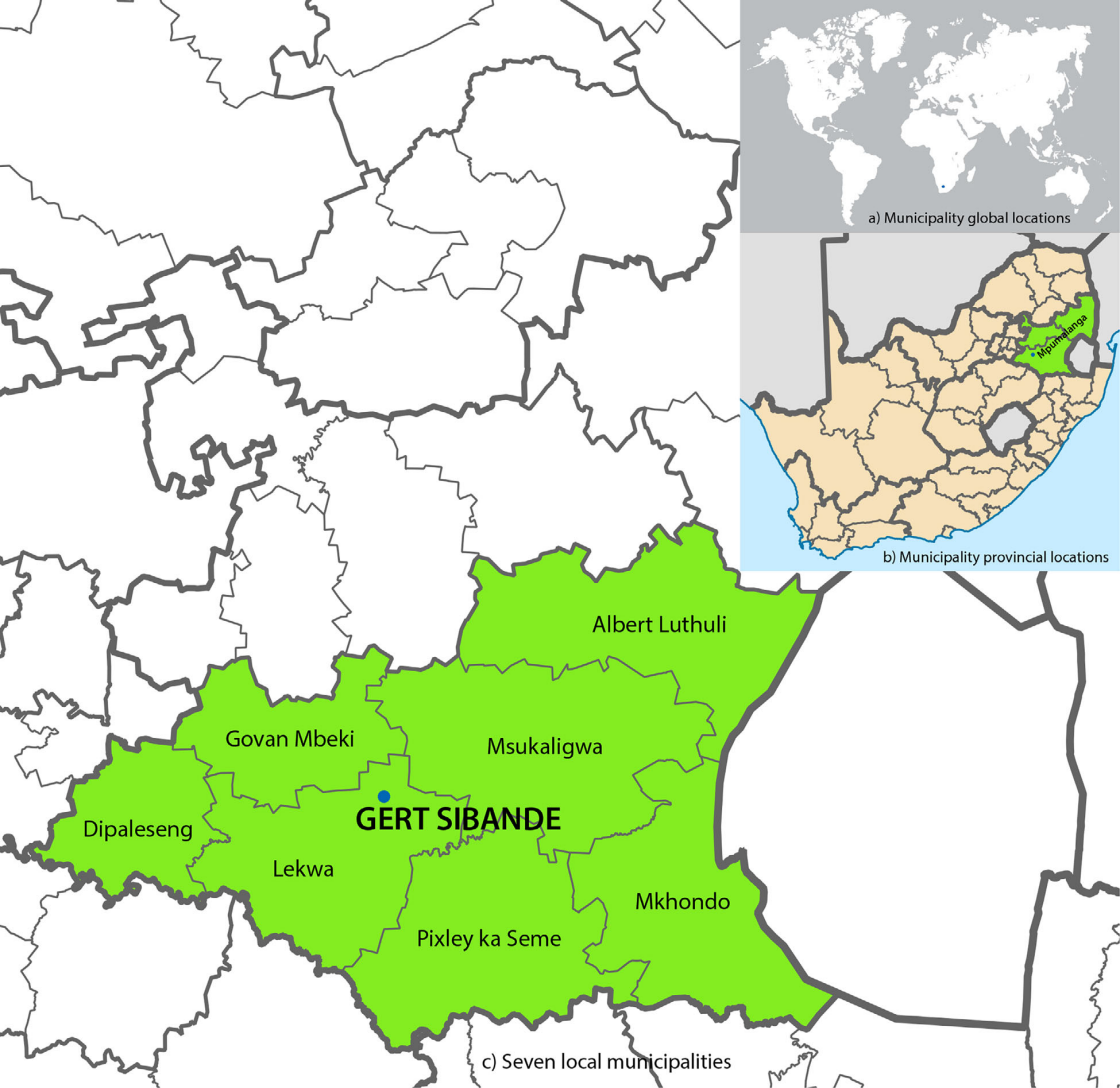
Geographical distribution of preschools within the Gert Sibande District Municipality. Part (a) provides a visual representation of the global location of the preschools within the Gert Sibande Municipality. Part (b) presents the spatial distribution of the Gert Sibande Municipality in the province of Mpumalanga within the broader context of the nine provinces of South Africa. (c) An illustration depicting the inclusion of all seven local municipalities within the Gert Sibande district, wherein preschools were identified.

The research study was carried out within the Mpumalanga Province, specifically in the Gert Sibande District Municipality. The Municipality is located within the Highveld Priority Area and was declared an air pollution priority area by the Minister of Environmental Affairs in 2007, per the National Environmental Management: Air Quality Act, 2004 (Act No. 39 of 2004). Under the Act, the Minister can declare an area, a priority area if the air in the area is deemed harmful to human health. The Highveld Priority Area has substandard air quality and heightened levels of pollutants originating from both industrial and non‐industrial origins. The district encompasses a diverse range of sectors, such as power generating, petrochemical, primary metallurgy, and open‐cast mining. The district municipality comprises seven local municipalities, specifically Dipaleseng, Govan Mbeki, Lekwa, Msukukaligwa and DR Pixley ka Seme, all of which are situated within the Highveld Priority Area. The Chief Albert Luthuli and Mkhondo municipalities are not encompassed under the Highveld Priority Area. See Figure 1.

### Sample size

NO_2_ and SO_2_ levels were measured indoors and outdoors at 13 preschools. These preschools were selected among the participants of a population‐based survey conducted between November 2020 and April 2021. The levels of NO_2_ and SO_2_ were measured indoors and outdoors from April 2022 to May 2022. There were 10 preschools (P1–P10) located in the Highveld Priority Area, and 3 preschools (P11–P13) located in a Non‐Highveld Priority Area. Three preschools, namely P5, P7 and P8, were situated close to a large road characterized by strong traffic. Additionally, three other preschools, P2, P4 and P9, were in suburban regions. This information is presented in Table 1. The selection of experimental locations in each preschool was based on practical considerations, including the safety of the samplers and the frequency of area occupancy. The placement of all rooms was on the ground floor. All the preschools were naturally ventilated via windows.

**TABLE 1:** This table lists the key characteristics of 13 preschools in the 7 districts of the Gert Sibande Municipality in Mpumalanga, South Africa that were chosen at random between April and May of 2022.

Preschool	District	Area description	Experimental areas
P1	Msukaligwa	Within the community area	The classroom and outside area
P2	Msukaligwa	Within the suburb area	The classroom and outside area
P3	Lekwa	Within the community area	The classroom and outside area
P4	Lekwa	Within the suburb area	The classroom and outside area
P5	Dipaleseng	Close distance to road with congested traffic	The classroom and outside area
P6	Dipaleseng	Within the community area	The classroom and outside area
P7	Govan Mbeki	Close proximity to street with heavy traffic	The classroom and outside area
P8	Govan Mbeki	Close proximity to street with heavy traffic	The classroom and outside area
P9	Dr Pixley ka Seme	Within the suburb area	The classroom and outside area
P10	Dr Pixley ka Seme	Within the community area	The classroom and outside area
P11	Chief Albert Luthuli	Within the community area	The classroom and outside area
P12	Chief Albert Luthuli	Within the community area	The classroom and outside area
P13	Mkhondo	Within the community area	The classroom and outside area

### Sampling method

#### Gaseous monitoring

**FIGURE 2:**
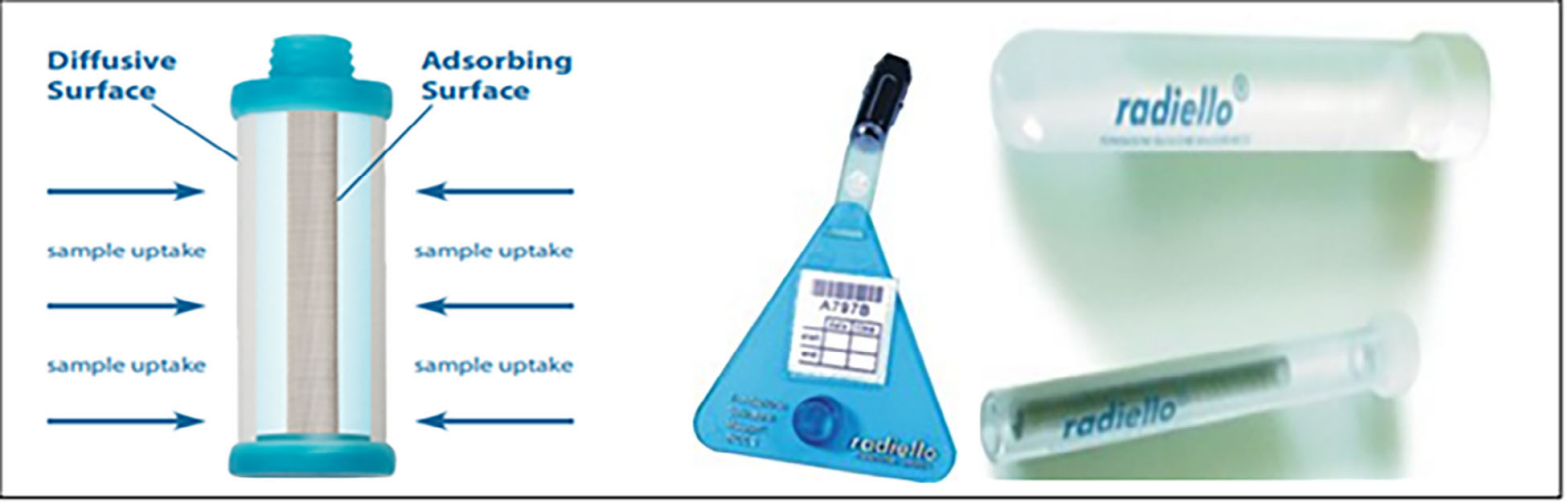
Radiello passive sampling system. *Source*: https://www.restek.com/global/en/articles/versatile‐high‐performing‐radiello‐passive‐air‐samplers.

Radiello combined SO_2_ and NO_2_ passive air samplers (RAD166) were used to measure indoor and outdoor SO_2_ and NO_2_ concentration levels [11]. Passive samplers require no electricity (expensive pumps), have no moving parts and are simple to use (no pump operation or calibration). Sampling was done continuously for 7 days, with sampling taking place 24‐h a day. The Radiello passive diffusion samplers have been thoroughly examined, validated and utilized by a multitude of researchers [12, 13]. The Radiello sampling system primarily consists of a blue microporous diffuse body; supporting plate, vertical adapter and an adsorption cartridge treated with triethanolamine in a sealed glass tube. Additionally,  an adhesive label with the bar code indication was included (Figure 2) [14]. The samples exhibited respective detection limits of 0.4 and 0.9 µg m^−3^. A total of 52 samples were gathered, with 26 collected from indoor locations and 26 from outdoor locations.

NO_2_ and SO_2_ levels were measured indoors and outside in each chosen classroom. To start with the sampling on the field, each cartridge was taken out of the tube and installed into the diffuse body without touching the cartridge. The body was then screwed onto the supporting plate. The tube and the stopper which were housing the cartridges were kept in the original plastic bag for use after sampling.

The samplers were assigned distinct labels, which were then placed in the pocket along with the date and time of the sampling. The indoor samplers were strategically placed at a height of approximately 1–1.5 m above the floor, precisely targeting the area where children breathe within the preschools. The selected place was positioned at a minimum distance of 1 m from all walls, doors and functioning heating systems. To carry out measurements in outdoor settings, the samplers were placed outside the preschools, at an elevation of 1.5—2 m from the ground. To guarantee their protection, the samplers were placed in a shelter and positioned at a minimum distance of 1 m from the closest structure. After the sampling process, the date and time of the sampling were recorded. Subsequently, the cartridge was reintroduced into the tube and duly labelled.

### Laboratory analysis

The NO_2_ and SO_2_ Radiello passive samplers were chemically analysed at laboratory SMBS Futura Dynamics in Pretoria, South Africa. This is an accredited laboratory specializing in the analysis of chemical substances that include air quality samplers. SO_2_ and NO_2_ Radiello passive samplers were analysed using sulphanilamide and *N*‐(1‐naphthyl)ethylenediamine dihydrochloride solutions, and the absorbance of the samples was measured through ion chromatography.

### Data entry and analysis

Qualtrics XM was used to enter and validate the data. The statistical analyses were conducted utilizing Version 17 of STATA. In total, 52 NO_2_/SO_2_ samplers were analysed (26 indoor and 26 outdoor). The data were given as the mean values over the 7‐day measurement period. Shapiro–Wilk tests were utilized to assess the presence of normality. As the distribution of air pollution levels was skewed, descriptive analysis employed non‐parametric tests. Separate Spearman rank correlation analyses were conducted for indoor and outdoor levels to ascertain the correlation coefficients among the various pollutants. The Wilcoxon paired sign rank test was utilized to determine whether or not there was a significant difference between the I/O ratios. The Wilcoxon–Mann–Whitney tests were utilized to determine whether or not there were significant differences between the I/O ratios and indoor and outdoor concentrations.

### Ethical considerations (IRB statement)

The present study received approval from the University of Pretoria Research Ethics Committee (Ethics Number: 766/2019), as well as the Gert Sibande District Municipality, Environmental Health Department and preschool principals. This paper presents only anonymized data.

## RESULTS

Table 3 provides detailed information regarding the levels of indoor and outdoor data, as well as the I/O ratios. The outdoor concentrations of the two pollutants were found to be lower than the South African Air Quality Standards, which specify a value of 200 mg m^−3^ for the 1‐h mean of NO_2_ and 125 µg m^−3^ for the 24‐h mean of SO_2_ (Table 2).

**TABLE 2:** World Health Organization (WHO) Air Quality Guidelines (AQG) and the South African Air Quality Standards.

Pollutants	WHO recommended guideline	South African Air Quality Standards
NO_2_	Interim target 1 40 µg m^−3^	
	Interim target 2 30 µg m^−3^	40 µg m^−3^ annual mean
	Interim target 3 20 µg m^−3^	200 µg m^−3^ 1‐h mean
	**AQG level 10 **µg m^−3^	
SO_2_	Interim target 1 120 µg m^−3^	125 µg m^−3^ 24‐h mean
	Interim target 2 50 µg m^−3^	500 µg m^−3^ 10‐min mean
	**AQG level 40 **µg m^−3^	50 µg m^−3^ annual mean

### NO_2_—indoor and outdoor

The average indoor NO_2_ concentration was somewhat lower compared to the outdoor levels (11.3 vs. 11.5 µg m^−3^) (Table 3). The current study found that the I/O ratios for NO_2_ varied from 0.40 to 2.90, as shown in Table 4. The average levels of NO_2_ indoors and outdoors were below the recommended criteria set by the WHO, specifically target levels 1–3. Additionally, these levels were also lower than the air quality guidelines (AQG) established by South Africa, both in terms of annual averages and 1‐h averages. Nevertheless, both levels remained higher than the recommended guideline of 10 µg m^−3^ set by the WHO AQG.

**TABLE 3:** Indoor and outdoor levels along with indoor−outdoor ratios at 13 randomly selected preschools from 7 districts in Mpumalanga Province, South Africa, April and May 2022.

Indoor	Mean	Median	SD	Range
NO_2(I)_	11.3	11.37	5.57	3.47–24.84
SO_2(I)_	1.38	0.56	0.07	0.07–5.03
Outdoor				
NO_2(O)_	11.5	11.30	6.19	3.38–25.34
SO_2(O)_	7.87	7.11	5.03	1.05–16.60
Indoor/outdoor ratios				
NO_2(I/O)_	1.22	1.10	0.60	0.40–2.90
SO_2(I/O)_	0.14	0.12	0.12	0.02–0.45

*Note*: Units for NO_2_ and SO_2_ in (µg m^−3^). *I/O ratio = C_in_/C_out_ (where C_in_ and C_out_ are the indoor and outdoor particle concentrations). Air quality sampling period – April–May 2022*.

**TABLE 4:** NO_2_ and SO_2_ indoor/outdoor ratios at 13 randomly selected preschools from 7 districts in Mpumalanga Province, South Africa, April and May 2022.

School	NO_2(I/O)_	SO_2(I/O)_
S1	1.28	0.12
S2	0.87	0.13
S3	0.89	0.10
S4	1.10	0.45
S5	0.76	0.25
S6	0.93	0.17
S7	1.18	0.30
S8	0.40	0.05
S9	1.02	0.03
S10	1.39	0.09
S11	1.75	0.04
S12	2.90	0.13
S13	1.42	0.02
**Mean I/O**	**1.22**	**0.14**
**Median I/O**	**1.10**	**0.12**
**Std I/O**		
**Range I/O**	**0.40–2.90**	**0.02–0.45**

### SO_2_—indoor and outdoor

The data for indoor SO_2_ exhibited non‐normal distribution, as indicated by the *p*‐values of 0.02 for the skewness and kurtosis test for normality, and *p*‐value of <0.001 for the Shapiro–Wilk test for normality. The average outdoor concentration of SO_2_ was 7.87 µg m^−3^, which was substantially more than the indoor concentration of 1.38 µg m^−3^. A significant disparity in SO_2_ levels was observed between outdoor and indoor environments, as determined by the Wilcoxon‐paired sign‐ranked test (*p*‐value = <0.001).

### Inter‐pollutant correlations

Comparing the two pollutants, the NO_2_ I/O ratios varied from 0.40 to 2.90, whereas the SO_2_ ratios ranged from 0.02 to 0.45. A notable difference was noted in the I/O ratios of the 2 pollutants among the 13 schools. The average I/O ratio for NO_2_ and SO_2_ exhibited a substantial difference, with a *p*‐value of <0.001. Additionally, the median I/O ratio for NO_2_ and SO_2_ showed a statistically significant difference, with a *p*‐value of <0.001, as determined by the Wilcoxon paired signed rank test.

## DISCUSSION

In this study, the outdoor mean concentrations of SO_2_ were consistently greater than the indoor concentrations in all of the preschools. Research conducted in Lisbon, Portugal, Taiyuan, China and Hong Kong, China, in the years 2011, 2008 and 2000, respectively, found comparable results for SO_2_ levels in schools, both indoors and outdoors [15, 16, 17]. Anticipated outcomes of the present investigation were projected to surpass both the WHO suggested benchmarks (targets 1–3) and the South African Air quality criteria, owing to the well‐established reputation of Mpumalanga Province for having the most unfavourable air quality in South Africa. The results must be interpreted with caution based on the fact that passive samplers were used to measure the air pollutants, and the results may underestimate the levels of gases in the environment. Future studies will have to employ direct reading instruments, which may be more accurate.

Moreover, the I/O ratios for NO_2_ in school environments have been reported to be within the range of 0.44–2.17 [18]. In a study conducted in the Mid‐Atlantic region, United States, the I/O ratios for NO_2_ in schools ranged from 0.5 to 3.1 [19]. The ratio of indoor‐to‐outdoor NO_2_ in school‐aged children might differ based on factors such as location, season and building characteristics. However, outdoor levels often serve as a dependable indicator of indoor NO_2_ levels throughout the year [20].

The respiratory health of school‐aged children can be significantly affected by exposure to both indoor and outdoor NO_2_, increasing the likelihood of developing asthma [21]. Indoor levels of NO_2_ can be influenced by elements such as gas stoves, heaters and tobacco smoke. On the other hand, outdoor levels of NO_2_ can be affected by sources, including traffic and industrial activities [21].

Research has indicated that children with asthma may experience negative respiratory effects due to exposure to NO_2_, and it has been observed that indoor levels of NO_2_ might be significantly greater than outside levels [22, 23]. Exposure to NO_2_ has been associated with respiratory symptoms and decreased lung function in children with asthma [21]. Efforts to reduce outdoor air pollution, such as traffic control measures, can help lower outdoor NO_2_ levels and protect the health of school‐aged children.

The outdoor concentration of SO_2_ was lower than the suggested recommendations set by the WHO (interim target 1, interim target 2 and the AQG level), as well as the Air Quality Standard in South Africa. Although the outdoor levels of SO_2_ are currently below or within the recommended guidelines, it remains crucial to consistently uphold a healthy air environment and minimize children's exposure to sulphur dioxide. Exposure to SO_2_ has been found to cause respiratory symptoms in children with asthma. Additionally, short‐term increases in SO_2_ levels resulting from emissions from refinery stacks have been associated with a greater number of asthma episodes in children living nearby [24].

Children with asthma, particularly, are sensitive to the harmful effects of SO_2_, which can make breathing difficult [25]. Controlled human exposure and epidemiological studies have shown that children with asthma are more likely to experience adverse responses to SO_2_ exposure compared to the non‐asthmatic population [26]. Long‐term studies have indicated possible associations between sulphur dioxide pollution and respiratory symptoms or reduced breathing ability in children [27]. These studies suggest that both indoor and outdoor SO_2_ levels can be associated with asthma in school children. However, more research is needed to fully understand the extent of the effects and to determine the best strategies for reducing children's exposure to SO_2_.

An advantage of the study is that passive sampling is generally comparable in accuracy to active sampling and eliminates the need for costly and impractical active sample equipment, such as pumps and flow meters. They exhibit cheap running expenses, possess a compact size and require no external power source [28]. The data were collected using passive sampling, capturing the mean concentrations over multiple days.

The study was limited by the inability to test the levels of NO_2_ and SO_2_ indoors and outdoors at the children's homes. This was due to parents/caregivers feeling uncomfortable and perceiving it as an invasion of privacy, maybe due to concerns about electronic surveillance. Passive samplers were used to measure the air pollutants which may underestimate the levels of gases in the environment. Future studies will have to employ direct reading instruments, which may be more accurate.

## CONCLUSION

Indoor and outdoor NO_2_ concentrations surpassed the WHO's recommended air quality standard. Exposure to air pollution is linked to an increased risk of developing respiratory disorders, particularly asthma. Hence, it is imperative to implement preventive measures to enhance the ventilation systems in preschools, employ air purifiers and eradicate potential sources of air pollutants.

## AUTHOR CONTRIBUTIONS

Rodney Mudau, Joyce Shirinde and Kuku Voyi participated in the study's design, Rodney Mudau was involved in the data collection and statistical analysis, and Rodney Mudau, Joyce Shirinde and Kuku Voyi were involved in interpreting the results and drafting and critically revising the manuscript. The published version of the work has been reviewed and approved by all authors.

## CONFLICT OF INTEREST STATEMENT

The authors declare no potential conflicts of interest concerning this article's research, authorship and/or publication.

## FUNDING INFORMATION

South African Medical Research Council's Division of Research Capacity Development, specifically through the Bongani Mayosi National Health Scholars Programme; the Public Health Enhancement Fund, South African National Department of Health

## INFORMED CONSENT STATEMENT

The present study received approval from the University of Pretoria Research Ethics Committee (Ethics Number: 766/2029), as well as the Gert Sibande District Municipality, Environmental Health Department, Department of Education, and preschool principals. This paper present only anonymized data.

## Data Availability

We did not receive ethics approval to share raw field data publicly. The data belong to the University of Pretoria (UP). The raw data analysed in the current study are available from UP on reasonable request.
